# Targeting the NLRP3 inflammasome and associated cytokines in scleroderma associated interstitial lung disease

**DOI:** 10.3389/fcell.2023.1254904

**Published:** 2023-10-02

**Authors:** Samuel Woo, Shifa Gandhi, Alexander Ghincea, Tina Saber, Chris J. Lee, Changwan Ryu

**Affiliations:** Department of Internal Medicine, Yale School of Medicine, Section of Pulmonary, Critical Care and Sleep Medicine, New Haven, CT, United States

**Keywords:** scleroderma associated interstitial lung disease, NLRP3, tumor necrosis factor, alpha, IL-1 beta, IL-18

## Abstract

SSc-ILD (scleroderma associated interstitial lung disease) is a complex rheumatic disease characterized in part by immune dysregulation leading to the progressive fibrotic replacement of normal lung architecture. Because improved treatment options are sorely needed, additional study of the fibroproliferative mechanisms mediating this disease has the potential to accelerate development of novel therapies. The contribution of innate immunity is an emerging area of investigation in SSc-ILD as recent work has demonstrated the mechanistic and clinical significance of the NLRP3 inflammasome and its associated cytokines of TNFα (tumor necrosis factor alpha), IL-1β (interleukin-1 beta), and IL-18 in this disease. In this review, we will highlight novel pathophysiologic insights afforded by these studies and the potential of leveraging this complex biology for clinical benefit.

## Introduction

SSc (scleroderma) is a complex rheumatic disease characterized by diffuse microvascular injury and immune dysregulation leading to the progressive fibrotic replacement of normal tissue architecture (Denton and Khanna, 2017). One of the leading causes of morbidity and mortality of this condition is the development of ILD (interstitial lung disease) (Volkmann and Fischer, 2021), where current therapeutic strategies include non-specific immunosuppressive drugs (Denton and Khanna, 2017) and anti-fibrotic agents (Distler et al., 2019) that are associated with heterogenous efficacy, high costs, and at times, intolerable side-effects (Rahaghi et al., 2023). Because better treatment options are sorely needed, additional study of the fibroproliferative mechanisms mediating this disease has the potential to accelerate development of novel therapies.

While the mechanisms by which autoimmunity engenders pathologic remodeling of the SSc lung remains poorly understood, aberrant adaptive immune responses and fibroblast activation are heavily implicated (Herzog et al., 2014). Although the contribution of innate immunity is less known in this context, its role in fibrogenesis has been an emerging area of investigation (Taroni et al., 2017). Innate immunity is activated by PRRs (pattern recognition receptors), which senses epitopes broadly conserved across pathogenic microbes, known as PAMPs (pathogen-associated molecular patterns), and endogenous ligands, known as DAMPs (danger-associated molecular patterns), that are released by stressed or activated cells and tissues (Ellson et al., 2014). When sensing excessive amounts of PAMPs or DAMPs, PRRs mediate activation of downstream inflammatory pathways (Li and Wu, 2021), including assembly of multimeric protein complexes known as inflammasomes, a caspase-1 activating platform (Franchi et al., 2009). While several PRRs are known to trigger inflammasome formation (Franchi et al., 2009; Kelley et al., 2019), the fibroproliferative role of the NLRP3 (NOD [nucleotide-binding oligomerization domain], LRR [leucine-rich repeat] containing proteins, and PYD-3 [pyrin domain-containing protein-3]) inflammasome has been of significant interest in SSc-ILD. Thus, the purpose of our review is to highlight recent work characterizing the mechanistic and clinical relevance of the NLRP3 inflammasome and its associated cytokines in this disease.

### NLRP3 inflammasome

Initially described in 2002, NLRP3 is a sensor protein that along with the adaptor protein ASC (apoptosis-related spot-like protein containing CARD [carboxy-terminal caspase recruitment domain]) and the effector protein caspase-1 comprises the NLRP3 inflammasome (Swanson et al., 2019; Yang et al., 2019). Its activation first requires a priming step mediated by NF-κB (nuclear factor kappa light chain enhancer of activated B cells) signaling, which occurs via PAMP or DAMP activation of TLRs (Toll-like receptors) or NOD2 (nucleotide binding oligomerization domain containing 2), or through soluble mediators such as TNFα (tumor necrosis factor alpha) and IL-1β (interleukin-1 beta) (Swanson et al., 2019; Gritsenko et al., 2020). Priming serves to (1) upregulate transcription of inflammasome components of NLRP3, pro-IL-18, and pro-IL-1β, and (2) induce post-translational modifications of NLRP3 to stabilize its function (Swanson et al., 2019; Gritsenko et al., 2020). Following this step, NLRP3 forms oligomers through homotypic interactions via NACHT domains (NAIP [neuronal apoptosis inhibitor protein], CIITA [MHC class II transcription activator], HET-E [incompatibility locus protein from *Podospora anserina*] and TP1 [telomerase-associated protein]) (Damiano et al., 2004), which recruits ASC proteins via homotypic PYD–PYD interactions to nucleate helical ASC filament formation (Swanson et al., 2019). Multiple ASC filaments coalesce into a single macromolecule, known as an ASC speck, to recruit caspase-1 through CARD–CARD interactions to facilitate caspase-1 self-cleavage and activation (Swanson et al., 2019; Yang et al., 2019). Caspase-1 cleaves and activates IL-1β, IL-18 (Swanson et al., 2019; Li et al., 2020), and the membrane pore-forming protein GSDMD (gasdermin-D), where its N-terminal domain (GSDMD^Nterm^) oligomerizes to create a cell membrane pore (Shi et al., 2015; Swanson et al., 2019). Though the exact mechanism of NLRP3 inflammasome activation remains elusive, many sources suggest that common second-messenger pathways link the multiple upstream signals to inflammasome activation, including potassium efflux, decrease in intracellular calcium, lysosomal disruption, mitochondrial dysfunction, and reactive oxygen species (Artlett and Thacker, 2015; Elliott and Sutterwala, 2015; Man and Kanneganti, 2015; He et al., 2016). Given the inflammatory properties inherent with NLRP3 inflammasome activation, this pathway has been interrogated in autoimmune conditions such as SSc-ILD.

Aberrant activation of the NLRP3 inflammasome has garnered significant interest for its potential pathogenic role in inflammatory conditions such as SSc-ILD. Genome-wide association studies have shown polymorphisms in the NLRP3 gene to be linked with the development of ILDs such as asbestosis (rs35829419) (Franko et al., 2020), coal workers pneumoconiosis (rs1539019) (Ji et al., 2012), and silicosis (rs1539019 and rs34298354) (Weng et al., 2015); while specific NLRP3 polymorphisms have yet to be identified in SSc-ILD, further study in this arena could identify convergent molecular mechanisms linking divergent disease states. However, translational work completed by Artlett and colleagues demonstrated the connection between NLRP3 inflammasome activation and SSc by showing that dermal fibroblasts from SSc patients exhibit increased expression of inflammasome components, and that experimentally induced caspase-1 inhibition of both dermal and lung SSc fibroblasts ameliorated collagen deposition, reduced IL-1β and IL-18 secretion, and decreased αSMA (alpha smooth muscle actin) expression (Artlett et al., 2011). Additionally, studies investigating the pathogenic contribution of chronic parvovirus B19 (B19V) infection in SSc revealed that monocytes derived from SSc patients express significantly elevated mRNA levels of *NLRP3* than monocytes derived from healthy control subjects in the presence of B19V infection (Zakrzewska et al., 2019). Furthermore, mice deficient in NLRP3 (−/−) and ASC (−/−) were shown to be resistant to BLM (bleomycin) induced skin and lung fibrosis (Artlett et al., 2011). Additional work validated the finding of NLRP3 overexpression in SSc, including the association between skin thickness and NLRP3 expression (Martinez-Godinez et al., 2015). More recently, it was shown that miR-155 (microRNA-155) expression is indispensable for NLRP3 inflammasome mediated collagen production in SSc dermal and lung fibroblasts (Artlett et al., 2017; Henderson and O'Reilly, 2017). NLRP3^−/−^ fibroblasts and those treated with caspase-1 inhibition had significantly decreased miR-155 expression and collagen synthesis, leading the authors to conclude that the NLRP3 inflammasome is required for miR-155 expression (Artlett et al., 2017; Henderson and O'Reilly, 2017). These discoveries not only advance our understanding of the pathophysiologic importance of the NLRP3 inflammasome in SSc, but also lend scientific rationale for targeting its inhibition.

Early preclinical studies targeting the NLRP3 inflammasome identified the sulfonylurea glyburide as a potential inhibiting agent as *in vitro* studies showed a reduction in caspase-1 activation and IL-1β and IL-18 secretion in ATP-treated, lipopolysaccharide-sensitized macrophages (Lamkanfi et al., 2009). MCC950 (also known as CRID-3 or CP-456,773) is a disulfonylurea compound similar to glyburide that has been shown to specifically bind to NLRP3 and prevent inflammasome activation, interrupting IL-1β, IL-1α, and IL-18 secretion in a myriad of preclinical work (Coll et al., 2015; Primiano et al., 2016; van der Heijden et al., 2017; van Hout et al., 2017; Perera et al., 2018; Coll et al., 2019; Tapia-Abellan et al., 2019; Vande Walle et al., 2019; Corcoran et al., 2021). While clinical evaluation of MCC950 led to its discontinuation due to hepatotoxicity (Chen et al., 2021), these early efforts provided the scientific premise for other small-molecule NLRP3 inhibitors that are in various phases of clinical trials, including dapansutrile, a specific NLRP3 inhibitor (Marchetti et al., 2018; Sanchez-Fernandez et al., 2019) that has been shown to be safe in heart failure (Wohlford et al., 2020) and gout (Kluck et al., 2020). Other small molecule NLRP3 inhibitors under investigation include IFM2427, inzomelid, somalix, IZD334, and NT-0167 (El-Sharkawy et al., 2020; Chen et al., 2021); their relevance and benefit to SSc-ILD will require additional translational and clinical evaluation.

Along with direct antagonism of the NLRP3 inflammasome, targeting soluble mediators related to this pathway has also been an area of active study. As stated above, TNFα and IL-1β mediate priming of the inflammasome, and its activation result in the production of IL-1β and IL-18. Moreover, integrative analysis of RNA sequencing studies of peripheral blood and lungs from SSc patients has further demonstrated the pathophysiologic significance of these cytokines (Kobayashi et al., 2021). Single cell RNA sequencing of lung tissue from SSc-ILD subjects revealed a subpopulation of monocytes (termed FCN1^hi^) that highly expressed, among others, genes related to *TNF, IL1B,* and *IL1R2* (Valenzi et al., 2019; Kobayashi et al., 2021). Thus, in the next section of this review, we will discuss the fibroproliferative contribution of TNFα, IL-1β, and IL-18 in SSc-ILD.

### Tumor necrosis factor alpha

TNFα is a pleiotropic pro-inflammatory cytokine produced by various stromal and immune cells that is initially expressed as a transmembrane precursor protein and undergoes cleavage by TACE (TNFα-converting enzyme) to release soluble TNFα (Black et al., 1997; Moha et al., 2002). Both transmembrane and soluble TNFα binds to TNFR1 (TNF receptor 1), while only its transmembrane form is able to recognize TNFR2 (TNF receptor 2) (Vandenabeele et al., 1995). TNFR1 is ubiquitously expressed and contains a conserved death domain that facilitates recruitment of the adaptor protein TRADD (TNFR1-associated death domain), triggering activation of four potential signaling complexes (Hsu et al., 1995). Complex I forms when TNF binds to TNFR1, leading to a conformation change in its cytoplasmic domain, leading to recruitment of key mediators that include TRADD, RIPK1 (receptor-interacting serine/threonine-protein kinase 1), TRAF2 (TNFR-associated factor 2), cIAP1/2 (cellular inhibitor of apoptosis protein 1 or 2), and LUBAC (linear ubiquitin chain assembly complex); these interactions mediate downstream events critical for canonical NF-κB and MAPK (mitogen-activated protein kinases) signaling that promote tissue and cell inflammation, survival, and proliferation (Baud and Karin, 2001; Micheau and Tschopp, 2003; Brenner et al., 2015). The formation of complexes IIa and IIb (known as apoptosomes) also involve TNF-TNFR1-TRADD-RIPK1 interactions, but also include recruitment of FADD (Fas-associated protein with death domain) and pro-caspase 8 to induce cytoplasmic apoptotic signaling; complex IIb also requires activation of RIPK3 (Cain et al., 1999; Wang et al., 2008; Brenner et al., 2015). Complex IIc (known as a necrosome) requires TNF-TNFR1-TRADD-RIPK1-RIPK3 interactions that mediate activation of MLKL (mixed lineage kinase domain-like protein) to initiate cellular necroptosis (Cho et al., 2009; Brenner et al., 2015). Meanwhile, TNFR2, expressed exclusively by immune and endothelial cells, lacks the death domain present in TNFR1, and alternatively recruits TRAF1 and TRAF2 to form Complex 1, leading to activation of NF-κB and MAPK pathways (Faustman and Davis, 2010; Brenner et al., 2015). Not surprisingly, TNFα signaling has been extensively evaluated in inflammatory conditions such as SSc-ILD.

Early translational studies with this cytokine demonstrated its critical role in experimental models of pulmonary fibrosis as TNFα was shown to modulate expression of TGFβ in various cells in the lungs (Warshamana et al., 2001; Sullivan et al., 2005; Sullivan et al., 2009). In elegant work completed by Sullivan and colleagues, they showed that both transcriptional and post-transcriptional modifications in TGFβ expression in mouse lung fibroblasts are induced by TNFα (Sullivan et al., 2005; Sullivan et al., 2009). In rodent models of lung fibrosis, mice exposed to BLM displayed increased expression of TNFα that was associated with TGFβ levels (Ortiz et al., 1998; Brass et al., 1999; Hou et al., 2018); moreover, adenoviral mediated TNFα overexpression in otherwise normal rat lungs resulted in upregulation of TGFβ1 and accumulation of αSMA expressing myofibroblasts (Sime et al., 1998). Additionally, in various murine models, genetic (Liu et al., 1998) and pharmacologic (Phan and Kunkel, 1992; Piguet et al., 1993) knockdown of TNFα and its receptor ameliorated chemically-induced lung fibrosis in a TGFβ dependent manner, suggesting a synergistic association between TNFα and TGFβ-mediated fibrosis. In a direct connection to the SSc disease state, high levels of TNFα have been detected in the BAL (bronchoalveolar lavage) (Bolster et al., 1997; Pantelidis et al., 2001) of SSc-ILD patients. Moreover, relative to alveolar macrophages (AMs) derived from SSc patients without pulmonary fibrosis, AMs from fibrotic SSc lungs displayed enhanced secretion of this cytokine (Pantelidis et al., 2001). However, other reports implicate an antagonistic relationship as chronic overexpression of TNFα not only protected mice from BLM-induced lung fibrosis (Fujita et al., 2003), but also accelerated resolution of this pathology through a reduction in profibrotic lung macrophages (Redente et al., 2014). This constellation of findings demonstrates a paradigm wherein TNFα exerts pathogenic or protective roles in pulmonary fibrosis that depends on cell-specific and temporal cues that would benefit from further evaluation.

Despite these findings, TNFα antagonism has been clinically therapeutic in inflammatory diseases such as RA (rheumatoid arthritis), ankylosing spondylitis, inflammatory bowel disease, and psoriasis, as well as various off-label indications (Haraoui, 2005; Jang et al., 2021). Not unexpectedly, this treatment strategy has been associated with exacerbating underlying ILD or resulting in *de novo* pneumonitis (Tengstrand et al., 2005; Andrew et al., 2006; Perez-Alvarez et al., 2011; Tamao et al., 2014), which has tempered enthusiasm for this agent in SSc-ILD. However, given that the blood of SSc patients is enriched for this cytokine (Pehlivan et al., 2012), particularly among those with lung disease (Murdaca et al., 2014), as well as the availability of several FDA-approved TNFα inhibitors, the indication for these agents in SSc-ILD have been explored. In an open-label pilot trial for diffuse cutaneous SSc, infliximab, a recombinant chimeric mouse/human monoclonal antibody, showed no significant improvement in MRSS (modified Rodnan skin scores); while there was potential for stabilization of skin disease, a high number of transfusion reactions limited use of this agent in this population (Denton et al., 2009). The role of infliximab remains unknown in SSc-ILD as there were no reports of its effect on lung function (Bosello et al., 2005; Denton et al., 2009); however, studies in sarcoidosis, another multi-system ILD, suggest that it has the potential to improve lung function (Baughman et al., 2006) and ameliorate multi-organ disease (Russell et al., 2013). Etanercept, a human TNF-receptor p75 Fc fusion protein that binds TNFα, showed promise in decreasing SSc associated inflammatory synovitis and was safely tolerated (Gordon et al., 2007). Although its role in SSc-ILD is largely unknown, in the same study, there was a no significant change in lung function (Gordon et al., 2007), and studies with this agent in sarcoidosis also demonstrated no change in lung function (Utz et al., 2003). Other FDA approved TNFα monoclonal antibodies include adalimumab and golimumab (Haraoui, 2005; Jang et al., 2021). While these agents have yet to be studied in SSc, they have been evaluated in sarcoidosis, where adalimumab has shown promise in improving lung function (Kamphuis et al., 2011; Milman et al., 2012; Sweiss et al., 2014), while golimumab demonstrated no benefit (Judson et al., 2014). Thus, despite the inflammatory nature of SSc-ILD, antagonism of TNFα signaling has demonstrated mixed results.

### Interleukin-1 beta

IL-1β is a cytokine mainly produced by myeloid cells in its inactive form, pro-IL-1β (Dinarello, 2011). It is cleaved intracellularly by caspase 1, and extracellularly by serine proteases that result in its activation (Lamkanfi, 2011; Bode et al., 2012). IL-1β binds to its receptor, IL-1R1, which heterodimerizes with the accessory protein IL-1RAcP (IL-1 receptor accessory protein) to transmit signaling via intracellular activation of adaptor proteins such as MyD88 (myeloid differentiation primary response gene-88), leading to the production of NF-κB and MAPK (Dinarello, 2009; Van Gorp et al., 2019; Chauhan et al., 2020). IL-1β signaling results in the production of additional pro-inflammatory cytokines and chemokines such as TNFα, IL-6, IL-8, MCP1 (monocyte chemoattractant protein 1), CXCL1 (chemokine ligand 1), and MIP2 (macrophage inflammatory protein 2) (Chauhan et al., 2020). IL-1β signaling also drives amplification and polarization of CD4^+^ T-cells toward a Th1 and Th17 phenotype and promotes differentiation of antigen-specific CD8^+^ T-cells (Ben-Sasson et al., 2009; Santarlasci et al., 2013; Chauhan et al., 2020). As an effector of both innate and adaptive immune responses, the study of IL-1β can lend novel immunopathogenic insight into inflammatory conditions such as SSc-ILD.

Somewhat surprisingly, studies of IL-1β signaling have led to conflicting findings. Initial translational efforts found that excessive concentrations of IL-1β were present in the lungs, blood, and skin of SSc patients (Hussein et al., 2005). In experimental models of lung fibrosis, mice treated with BLM displayed increased IL-1β production, inflammation, remodeling, and fibrosis in a manner dependent on IL-1R1/MyD88 signaling (Gasse et al., 2007). These findings were significantly reduced in IL-1R1-and MyD88-deficient mice, suggesting the indispensable nature of endogenous IL-1β in pulmonary inflammation and fibrosis (Gasse et al., 2007). However, these findings are tempered by recent reports showing that IL-1 receptor inhibition can exacerbate pulmonary fibrosis; employing a murine model of SSc through the overexpression of Fra-2 (fos-related antigen-2), mice treated with an IL-1 inhibitor demonstrated worse lung function, enhanced Th2 inflammation, and greater numbers of pro-fibrotic, alternatively activated macrophages (Birnhuber et al., 2019). Similar to that of TNFα, these data demonstrate the context-dependent nature of IL-1β that warrants additional investigation.

Nonetheless, re-purposing FDA-approved IL-1β antagonists has been considered for this and other autoimmune diseases. Anakinra is a recombinant human IL-1Ra that competitively inhibits IL-1α and IL-1β interaction with IL-1R1 and is currently indicated for the treatment of refractory RA and for CAPS (Cryopyrin Associated Periodic Syndrome) (Cavalli and Dinarello, 2015). Its safety and efficacy have yet to be explored in SSc. Canakinumab is a human monoclonal anti-IL-1β antibody that has demonstrated efficacy in treating multiple autoimmune conditions (De Benedetti et al., 2018) as well atherosclerotic diseases in the CANTOS Trial (Ridker et al., 2012; Ridker et al., 2017a). Interestingly, additional analysis of the CANTOS cohort revealed a significant reduction in incident lung cancer and lung cancer mortality (Ridker et al., 2017b); these findings suggest that this agent is able to modulate processes in the lung, making it a promising candidate for treating SSc-ILD. Lastly, rilonacept is a soluble IL-1 trap that binds IL-1α and IL-1β; it has been approved for the treatment of CAPS (Hoffman et al., 2012) and has demonstrated safety and efficacy in other inflammatory disorders (Krause et al., 2012). Moreover, in a small cohort of SSc patients (*n* = 19), this drug, while demonstrating an acceptable safety and tolerability profile, did not demonstrate biologic (based on gene expression) or clinical (based on MRSS) efficacy (Manter et al., 2018). Ultimately, further work is required to determine the clinical benefit of IL-1β inhibition in SSc-ILD.

### Interleukin-18

Along with IL-1β, NLRP3 inflammasome activation mediates production of IL-18, formerly known as IFN-γ (interferon-gamma) inducing factor. This cytokine is produced as pro-IL-18 by a number of cells, including macrophages and dendritic cells, and as with IL-1β, requires cleavage by caspase-1 to become biologically active (Arend et al., 2008; Dinarello et al., 2013). The IL-18 receptor, comprised of IL-18Rα and IL-18Rβ subunits, forms a high-affinity heterodimer expressed on various immune, endothelial, and smooth muscle cells, and is modulated by various other cytokines (Arend et al., 2008). Similar to the IL-1 receptor, IL-18 signal transduction proceeds via several intracellular adaptor molecules, including MyD88, IRAK1 (interleukin 1 receptor associated kinase 1), and TRAF6 to activate MAPK, NF-κB, and JNK (c-Jun N-terminal kinase) (Arend et al., 2008; Dinarello et al., 2013; Xu et al., 2019; Yasuda et al., 2019). IL-18 stimulates production of GM-CSF (granulocyte-monocyte colony-stimulating factor), TNFα, and IL-1β, and mediates both innate and adaptive immune responses (Arend et al., 2008). In the presence of IL-12 and IL-15, IL-18 can induce IFN-γ production by various T-cells and favors Th1 differentiation, a pro-inflammatory state; in the absence of IL-12 and IL-15, IL-18 signaling favors Th2 differentiation and promotes fibrosis (Dinarello et al., 2013; Kaplanski, 2018). Much like IL-1β, IL-18 has pleotropic effects in immune activation, suggesting a potential contribution in inflammatory conditions such as SSc-ILD.

Similar to work done with TNFα and IL-1β, studies of IL-18 have also led to contradictory results in SSc-ILD. In the serum of patients with SSc, increased concentrations of IL-18 were identified, and these levels negatively correlated with lung function, suggesting a role in mediating lung disease (Lin et al., 2019). However, both profibrotic and anti-fibrotic properties of IL-18 have been illustrated. In experimentally-induced pulmonary fibrosis, mice subjected to BLM administration exhibited increased concentrations of IL-18 in the lung (Oku et al., 2008). Additionally, novel work investigating the myeloid-specific contribution of autophagy in lung injury showed that mice deficient in the key autophagy gene *Atg7* develop spontaneous lung inflammation that was predominantly mediated by IL-18 from constitutive inflammasome activation (Abdel Fattah et al., 2015). Here, the authors showed that IL-18 antagonism inhibited the recruitment of lymphocytes and neutrophils in the lungs of these mice, suggesting a critical role for myeloid-specific IL-18 (Abdel Fattah et al., 2015). However, another study showed that mice deficient in IL-18 exhibited greater lung injury and weight loss in response to BLM administration (Nakatani-Okuda et al., 2005). In that same study, while therapeutic administration of IL-18 had no effect on BLM-induced lung injury, prophylactic IL-18 treatment prior to BLM administration ameliorated findings of lung injury (Nakatani-Okuda et al., 2005). The pleotropic effects of IL-18 altering the balance between Th1 and Th2 responses is likely contributing to this conundrum (Kim et al., 2010), and further study is sorely needed to unravel this complex biology.

Despite these conflicting *in vivo* data, IL-18 inhibition is currently under investigation for therapeutic benefit. Tadekinig alfa, a recombinant human IL-18 binding protein, was shown to be effective in treating adult onset Still’s disease (Gabay et al., 2018; Kiltz et al., 2020) and NLRC4 associated macrophage activation syndrome (Canna et al., 2017). GSK1070806, a monoclonal IL-18 antibody, was shown to be safely tolerated (Mistry et al., 2014) and is currently being evaluated in a phase 1b clinical trial for atopic dermatitis (NCT04975438) and phase 2 clinical trial for moderate to severe Crohn’s disease (NCT03681067). Additional work will be needed to determine whether IL-18 is promoting disease or protecting the lung in SSc-ILD.

## Conclusion

**FIGURE 1:**
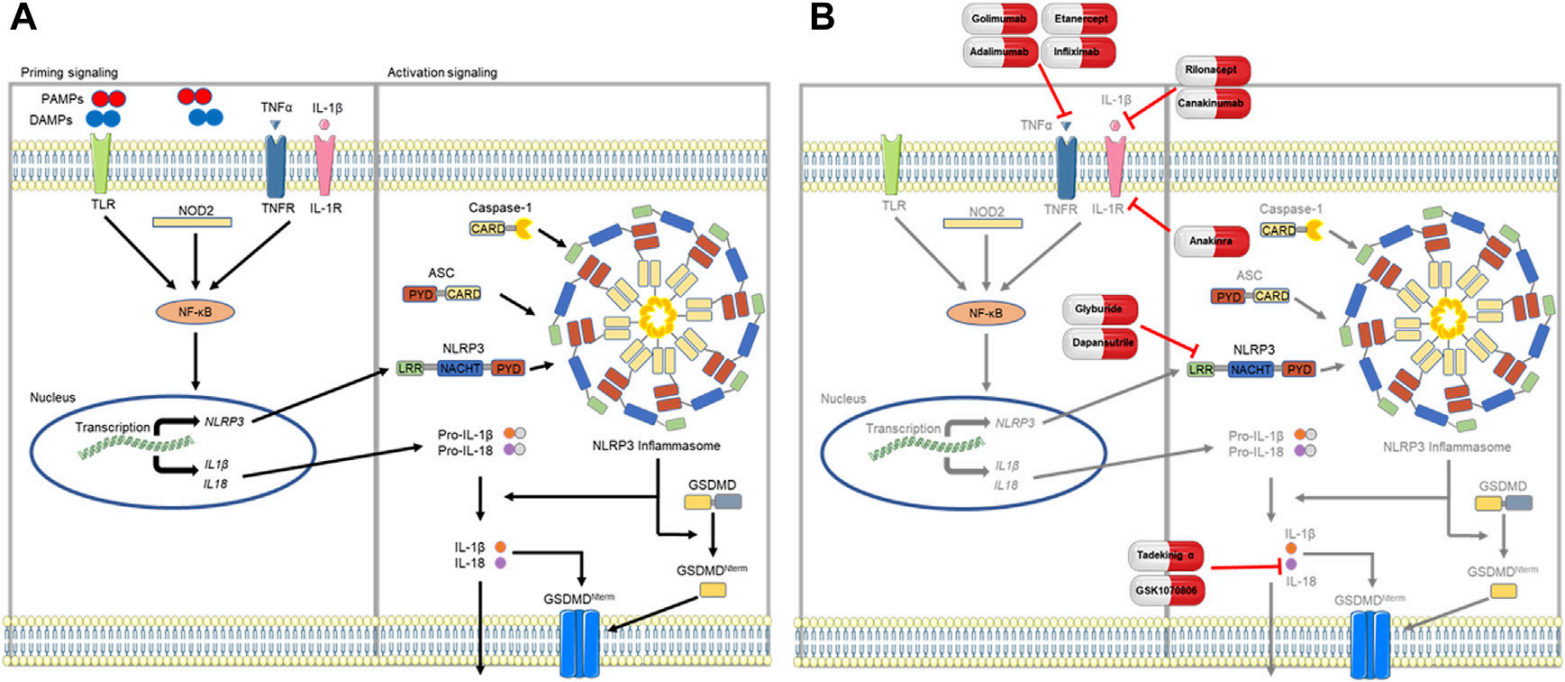
Proposed model of NLRP3 inflammasome signaling. **(A)** Assembly of the NLRP3 inflammasome first requires a priming step that is mediated by NF-κB (nuclear factor kappa light chain enhancer of activated B cells) signaling, which occurs via PAMP (pathogen associated molecular pattern) or DAMP (damage associated molecular pattern) activation of TLRs (toll-like receptors) or NOD2 (nucleotide binding oligomerization domain containing 2), or through soluble mediators such as TNFα (tumor necrosis factor alpha) and IL-1β (interleukin-1 beta). Priming upregulates transcription of inflammasome components of NLRP3, pro-IL-18, and pro-IL-1β. Following this step, components of the NLRP3 inflammasome coalesce to form the ASC speck and subsequently the inflammasome, activating caspase-1 to cleave pro-IL-1β and pro-IL-18 into their respective products, IL-1β and IL-18. **(B)** Antagonism of the NLRP3 inflammasome or mediating cytokines (TNFα, IL-1β, and IL-18) have shown potential as novel therapeutic targets in SSc-ILD. Parts of the figure were drawn by using pictures from Servier Medical Art. Servier Medical Art by Servier is licensed under a Creative Commons Attribution 3.0 Unported License (https://creativecommons.org/licenses/by/3.0/).

This concludes our review of NLRP3 inflammasome signaling and associated cytokines of TNFα, IL-1β, and IL-18 as novel therapeutic targets in SSc-ILD; these findings are summarized in Table 1 and depicted in Figure 1. Study of this complex biology requires further investigation to fully characterize the context-dependent roles of this signaling pathway and soluble mediators in protecting against or promoting pulmonary fibrosis in SSc-ILD. Addressing such knowledge gaps require improved modeling systems that better recapitulate human disease; rodent models exhibit relatively swift progression of lung fibrosis, fail to reproduce lung histopathology, and resolve fibrosis with discontinuation of the pro-fibrotic agent (Herzog et al., 2014). Thus, while animal modeling is viewed as a useful tool for the *in vivo* study of lung disease that would be unethical and impractical in humans, their value is limited. Work complemented by studies of primary human cells and tissues, as well as functional studies of explanted cells and organs, have been instrumental in replicating the salient features of the healthy and diseased adult mammalian lung (Herzog et al., 2014). Translational studies integrating traditional *in vivo* models with state-of-the-art *ex vivo* mimetics will have more direct relevance to the human disease state. Such work has the potential to yield insight into novel pathophysiologic mechanisms while catalyzing new treatment approaches, including repurposing old and developing new drugs, in this intractable condition.

**TABLE 1:** Antagonists and their mechanism of action.

Antagonist	Mechanism of action
Adalimumab	• Recombinant human IgG1 anti-TNFα monoclonal antibody
	• Inhibits binding of both soluble and transmembrane TNFα to TNFα receptors
Anakinra	• Recombinant non-glycosylated human IL-1 receptor antagonist
	• Competitively inhibits IL-1 binding to the interleukin-1 type I receptor
Canakinumab	• Recombinant human IgGκ anti-IL-1β monoclonal antibody
	• Binds to IL-1β to inhibit IL-1 receptor activation
Dapansutrile	• β-sulfonyl nitrile compound
	• Binds to the NLRP3 protein to prevent NLRP3 ATPase activity and NLRP3-ASC interactions
Etanercept	• Soluble TNF receptor
	• Binds to both TNFα and TNFβ to inhibit TNF receptor activation
Golimumab	• Human IgG1κ anti-TNFα monoclonal antibody
	• Binds to soluble and transmembrane TNFα to inhibit TNFα receptor activation
Glyburide	• Sulfonylurea compound
	• Unknown mechanism of action, believed to inhibit potassium efflux needed for NLRP3 inflammasome activation
GSK1070806	• Human IgG anti-IL-18 monoclonal antibody
	• Binds to IL-18 to inhibit IL-18 receptor activation
Infliximab	• Chimeric IgG1 anti- TNFα monoclonal antibody
	• Binds to soluble and transmembrane TNFα to inhibit TNFα receptor activation
Rilonacept	• Soluble IL-1β receptor
	• Binds to IL-1β to inhibit IL-1 receptor activation
Tadekinig alpha	• Recombinant IL-18 binding protein
	• Binds to free IL-18 to inhibit IL-18 receptor activation
